# Redfield Ratios in Inland Waters: Higher Biological Control of C:N:P Ratios in Tropical Semi-arid High Water Residence Time Lakes

**DOI:** 10.3389/fmicb.2017.01505

**Published:** 2017-08-08

**Authors:** Ng H. They, André M. Amado, James B. Cotner

**Affiliations:** ^1^Graduate Program in Ecology, Limnology Laboratory, Department of Oceanography and Limnology, Universidade Federal do Rio Grande do Norte Natal, Brazil; ^2^Department of Biology, Universidade Federal de Juiz de Fora Juiz de Fora, Brazil; ^3^Department of Ecology, Evolution, and Behavior, University of Minnesota, St. Paul MN, United States

**Keywords:** nutrients, metabolism, bacteria, ecological stoichiometry, semi-arid, tropical, lakes, water residence time

## Abstract

The canonical Redfield C:N:P ratio for algal biomass is often not achieved in inland waters due to higher C and N content and more variability when compared to the oceans. This has been attributed to much lower residence times and higher contributions of the watershed to the total organic matter pool of continental ecosystems. In this study we examined the effect of water residence times in low latitude lakes (in a gradient from humid to a semi-arid region) on seston elemental ratios in different size fractions. We used lake water specific conductivity as a proxy for residence time in a region of Eastern Brazil where there is a strong precipitation gradient. The C:P ratios decreased in the seston and bacterial size-fractions and increased in the dissolved fraction with increasing water retention time, suggesting uptake of N and P from the dissolved pool. Bacterial abundance, production and respiration increased in response to increased residence time and intracellular nutrient availability in agreement with the growth rate hypothesis. Our results reinforce the role of microorganisms in shaping the chemical environment in aquatic systems particularly at long water residence times and highlights the importance of this factor in influencing ecological stoichiometry in all aquatic ecosystems.

## Introduction

Life has had a strong impact in influencing the availability of elements on the Earth. About 2.3 billion years ago the atmosphere was drastically changed to an oxidative state with the rise of O_2_ and fall of CO_2_ concentrations that were preceded by the ‘invention’ of photosynthesis (Kasting and Siefert, 2002). As the redox state of the Earth changed, organisms had to adapt to the changing steady state. Similar processes occur in the oceans today, whereby micro-organisms alter their external chemical environment, which is reflective of their own elemental biomass composition, i.e., stoichiometry. The mean elemental composition of ocean plankton with respect to the macroelements carbon (C), nitrogen (N) and phosphorus (P) was first discussed by Redfield (1934, 1958) and the ratio of 106C: 16N: 1P is referred today as the ‘Redfield ratio.’

In marine waters, it is now clear that there is variability around the Redfield ratio due to latitudinal changes and nutrient availability effects (Martiny et al., 2013), whereas in inland waters the C:N:P ratios are often higher in magnitude and with greater variability (Hecky et al., 1993; Hassett et al., 1997; Sterner et al., 2008). The seston C:N:P ratios are influenced by species composition (e.g., eukaryotes *vs.* prokaryotes, N-fixing cyanobacteria, diatoms, etc.), temperature, organic matter inputs (quality and quantity), light:nutrient ratio, total P, chlorophyll, POC (particulate organic carbon): chlorophyll ratio and water residence time (WRT) (Hecky et al., 1993; McGroody et al., 2004; Hessen, 2006; Geider and La Roche, 2002; Martiny et al., 2013). Particularly in inland waters, it is often assumed that the income of large amounts of organic matter from terrestrial environments with typically high C:N:P ratios, as well as a variable share of phytoplankton (autochthonous production) relative to total seston (Hessen et al., 2003), and a tendency of lower environmental stability in space and time could cause the great variability in the seston elemental ratios when compared to the oceans (but see Hessen et al., 2003 and Hessen, 2006).

The residence times of substances in the oceans are quite long. The water has a residence (WRT) time of >3,000 years on average, while NO_3_^-^ and PO_4_^3-^ have a much longer residence time (∼10^4^ years) (Falkowski and Davis, 2004). In contrast, in inland waters the WRT can be as low as days to 10s of 1000s years (Bell et al., 2002), but are typically much shorter than in the oceans. The residence times of elements such as P are comparable to WRT, usually less than a year to a few years (Sonzogni et al., 1976). Nitrogen retention time also is highly dependent on WRT, but has a more complex cycle and its retention depends on the type and abundance of vegetation, nitrification and denitrifications rates (Saunders and Kalff, 2001). Even though it has been debated about the general applicability of the classical Redfield ratio in the oceans (Koeve and Kähler, 2010; Martiny et al., 2013) and organisms (Geider and La Roche, 2002), the central tenet of Redfield’s idea remains valid today (Falkowski and Davis, 2004; Gruber and Deutsch, 2014).

Microbes including virus, archaea, autotrophic and heterotrophic bacteria and algae play a central role in shaping aquatic ecosystem nutrient ratios (e.g., C, N, and P) and bacteria are the major consumers of P and N-based compounds in both marine and inland waters (Kirchman, 1994; Cotner and Biddanda, 2002). Nutrient concentrations and availability in the water column in turn also affect bacteria through constraints on metabolism and biomass composition. Bacteria growing under P-rich conditions (expressing high growth rates) are expected to exhibit low biomass C:P. High growth rates are associated with major allocation of P for nucleic acids in ribosomes, which is known as the Growth Rate Hypothesis (GRH) (Elser et al., 1996). Conversely, when growing under P-limiting conditions bacteria may either present high C:P ratios in the biomass (Cotner et al., 2010; Godwin and Cotner, 2015) or exhibit high respiration rates (Cimbleris and Kalff, 1998), possibly as a mechanism to eliminate the excess carbon from organic matter and to concentrate the P to an adequate amount (Hessen and Anderson, 2008). However, bacteria can also take up inorganic nutrients directly from the water (Cotner and Wetzel, 1992; Kirchman, 1994), which could possibly explain a compensation for low quality (high C:nutrients) substrates.

Most studies on ecological stoichiometry have examined the whole seston rather than examining the different planktonic groups (e.g., phytoplankton and bacterioplankton size fractions) separately and usually ignore the dissolved organic matter fraction (but see Hessen et al., 2003). For instance, the dissolved organic matter fraction and bacteria fraction are not well studied despite accounting for the largest part of the organic matter in many aquatic ecosystems (Wetzel, 1984; Simon et al., 1992; Gasol et al., 1997). Moreover, differences are expected in terms of stoichiometric flexibility (ability to adjust the internal C:N:P compositions in relation to the sources) among the planktonic components. The zooplankton have been reported to be quite inflexible, (Andersen and Hessen, 1991; Persson et al., 2010; Godwin and Cotner, 2015) and the phytoplankton community is more variable and more flexible (Geider and La Roche, 2002), responding to a wide gradient of light:nutrients ratios (Sterner et al., 1997; Hessen et al., 2002). However, a recent study has shown that the bacterial community can be the most flexible group of heterotrophic organisms known so far (Godwin and Cotner, 2015), with a high potential for adjusting and affecting environmental nutrient availability. The dissolved organic nutrient pools can buffer fluctuations in planktonic nutrient requirements depending on the rate of supply from external environments and their nutrient ratios. Heterotrophic bacteria grow relatively fast and consume primarily dissolved pools of inorganic and organic matter and thus are an important factor determining the elemental stoichiometry of both the dissolved and particulate pools.

The great variability in residence time in inland water ecosystems relative to oceans may be a key feature explaining the departure of freshwaters from Redfield ratios, i.e., a higher decoupling between bacteria and their substrates in inland water ecosystems due to variable WRT. To examine this hypothesis, we tested the effect of increasing WRT on the stoichiometry of seston, bacterial and dissolved fractions, as well as on bacterial metabolism in low latitude coastal and semi-arid lakes encompassing a wide range of trophic states and WRT. The sampling of aquatic systems in Eastern Brazil coincided with a strong El Niño event that began in June 2014 and extended through 2016.^1^ This phenomenon is associated with droughts in Northeastern Brazil, particularly in the semi-arid zone (Rodrigues et al., 2011). Semi-arid lakes in eastern Brazil may experience extended periods of drought and high rates of water evaporation. Since these semi-arid lakes receive only the contribution of intermittent rivers, the inflow rates are negligible (close to zero) during drought, particularly for severe episodes such as the El Niño period that occurred during this study. Hence, drought has then an indirect effect on increasing WRT. This is paralleled by increasing concentrations of nutrients and increased specific conductivity, which in turn may be used as a proxy for WRT (Curtis and Adams, 1995; Rennella and Quirós, 2006; Anderson and Stedmon, 2007).

## Materials and Methods

### Study Area

We sampled 15 ecosystems that consisted of both natural lakes and artificial reservoirs located in northeastern Brazil in Rio Grande do Norte state that encompass a trophic gradient ranging from oligotrophy to hypereutrophy and a wide range of environmental conditions (**Table 1**). These ecosystems are found in a relatively narrow latitudinal (from 06°24′36.5′′ S to 06°41′42.9′′ S) and longitudinal (from 35°05′59.4′′ W to 36°37′43.6′′ W) range that, nonetheless encompass a strong climatic gradient from the humid coastal zone (oligo- to mesotrophic lakes) to the inner, semi-arid zone (mostly eutrophic lakes and with longer WRT).

**Table 1:** Main limnological characteristics of the 15 low latitude lakes sampled along a trophic gradient.

Lake	Location	Sampling point depth	Secchi	Temp.	pH	Alkalinity	Conductivity	Chla	TP	DOC:TDP
		(m)	(cm)	(°C)		(μEq L^-1^)	(mS cm^-1^)	(μg L^-1^)	(μM)	(mol: mol)
Bonfim	06°02′18.4′′S/35°12′50.0′′W	11.8	580	28.23	5.7	90.0	0.090	0.24	0.96	104
EAJ	05°53′23.3′′S/35°21′32.6′′W	6.8	308	28.85	6.1	441.6	0.095	0.24	0.68	1938.3
Extremoz	05°42′45.1′′S/35°17′06.3′′W	4.25	230	28.56	6.7	960.1	0.162	1.08	0.51	382.7
Arituba	06°04′42.3′′S/35°06′17.9′′W	2.4	240	28.66	5.7	5.1	0.066	1.87	0.60	311.1
Carcará	06°03′41.2′′S/35°09′35.4′′W	3.9	300	28.29	5.7	25.9	0.040	2.07	0.57	490.8
Jiqui	05°55′08.3′′S/35°11′14.0′′W	3.0	230	29.79	6.2	287.7	0.073	2.33	0.68	121.8
Lagoa Azul	05°42′48.2′′S/35°15′56.6′′W	2.55	69	28.03	6.6	717.7	0.174	3.25	1.21	389.1
Jambeiro	05°52′06.3′′S/35°20′25.2′′W	6.0	81	29.34	6.7	1473.0	0.200	2.03	0.85	811.7
Ilhota	05°59′26.7′′S/35° 07′34.1′′W	1.3	130	29.22	5.9	50.5	0.045	5.93	0.68	332.3
Cruzeta	06°24′36.5′′S/35°47′44.4′′W	3.1	56	26.86	8.1	3923.2	0.353	6.27	2.52	444.1
Caliman	06°04′00.1′′S/35°05′59.4′′W	7.3	73	29.18	6.5	840.0	0.129	6.27	1.01	801.1
Boqueirão	06°41′42.9′′S/36°37′43.6′′W	9.9	45	25.40	8.4	7576.5	2.364	8.05	1.04	6259.8
Gargalheiras	06°25′30.6′′S/36°36′07.9′′W	10.4	33	28.40	8.5	6795.3	1.400	13.42	4.49	1886.7
Passagem das Traíras	06°30′52.6′′S/36°56′32.2′′W	5.1	20	28.14	8.7	10967.4	2.496	24.40	2.77	2925.4
Dourado	06°14′47.2′′S/36°30′33.8′′W	2.0	14	24.99	7.8	3770.1	1.133	44.53	5.80	1175.3

*Temp, temperature; Chla, chlorophyll *a*; TP, total phosphorus; DOC, dissolved organic carbon (TOC < 0.7 μm); TDP, total dissolved phosphorus (total phosphorus < 0.7 μm)*.

The coastal region climate is classified as humid with average annual temperature above 26°C. The historical annual average precipitation is 1,230 mm with 90% of rainfall between January and August. The vegetation in this area is classified as Atlantic rain forest (see Cestaro and Soares, 2004), which contributes with significant organic matter to aquatic ecosystems in the rainy season (Kosten et al., 2010). The inner region climate is classified as tropical and semi-arid (BS’h’ according to Kottek et al., 2006) with annual average temperature higher than 25°C. It is characterized by irregular rainfall, high evapotranspiration rates, and negative water balance during most of the year, particularly in the last 3 years. The mean annual precipitation is 733 mm in the previous 20 years and the aquatic ecosystems have long residence times (e.g., water residence time calculated in 2004 from 780 days in Boqueirão reservoir to 1460 days in Gargalheiras reservoir, both sampled in the current study; Chellappa et al., 2009). However, the annual precipitation was below the average (between 200 and 600 mm per year) and the aquatic ecosystems have experienced extreme droughts since 2012 (Costa et al., 2016). Because of that, these ecosystems have experienced extreme reductions in volume (Medeiros et al., 2015).

### Sampling

Each lake was sampled once close to its central point between October and December in 2014. In each lake, 10 L of sub-surface water (20–30 cm) was collected in acid-rinsed (HCl 10%) polyethylene bottles. The water transparency was determined with a secchi disk and the temperature and conductivity were measured *in situ* using a multi-parameter probe (Horiba U-22). The depth of the sampling point was determined with a depth meter or manually with a weight tied to a rope. Two subsamples were taken in plastic bottles without headspace for the determination of alkalinity. All samples were processed within 24 h; when the time between sampling and handling exceeded 4 h, the samples were kept refrigerated.

### Limnological Variables and Fractionation

**FIGURE 1:**
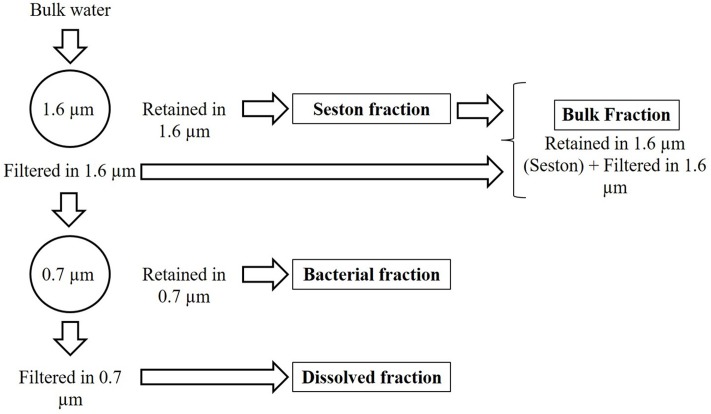
Scheme of the size fractionation of the water.

In the laboratory, the pH (Hanna HI-221) and alkalinity by Gran titration (H_2_SO_4_ 0.0125 M) were immediately measured. The water was fractionated by sequential filtration through 1.6 μm mean retention pore-size glass fiber filters (Whatman GF/A) and 0.7 μm mean retention pore-size glass fiber filters (Whatman GF/F). This procedure was adopted to separate the seston (here assumed as > 1.6 μm), bacterial (here assumed as particles between 1.6 and 0.7 μm) and dissolved fractions (here assumed as < 0.7 μm). The bulk fraction was estimated as the sum of seston (>1.6 μm) and the <1.6 μm fraction (**Figure 1**). The bacterial fraction possibly includes archaeal cells, but since Archaea are in general a minor component (<6%) of pelagic prokaryote communities (Glöckner et al., 1999), we refer to this fraction as bacteria. The 1.6–0.7 μm fraction included on average 79% of the bacteria. Even though the 0.7 μm filter allowed some bacteria to pass, the glass fiber filter can be used for the analysis in the TOC equipment without interference of the filter components (such as C and N from 0.2 μm porosity membrane filters often made of C, N and P-containing polymers) and has been successfully employed in similar studies for the same purpose (Cotner et al., 2010). The bacterial density (BD) was determined in the bulk, bacterial and dissolved fractions (see below), and appropriate corrections were applied by estimating mean bacterial cell C, N, and P content in the bacterial fraction and summing up the nutrients amounts in the retained or filtered bacteria (Supplementary Table 1). It is noteworthy to mention that our understanding of the stoichiometry of bacteria is somewhat biased by the fact that we did not have appropriate tools for distinguishing the composition and abundance of free and attached bacteria, with the last being part of the seston. This should have a little impact on the results found, since typically attached bacteria represent a small proportion (<30%) of the total bacterial pool (Simon, 1987).

Chlorophyll *a* was measured after filtration of bulk water through a 1.2 μm mean retention pore size glass fiber filter (GF/C Whatman). The filters were kept at -80°C and the chlorophyll *a* was extracted with ethanol (90%) at -20°C in the dark (Nusch and Palme, 1975; APHA, 1999). The chlorophyll was measured without acidification at 665 nm after correction for turbidity (750 nm) and calculated according to Salonen and Sarvala (1995) and Schilling et al. (2006).

### C, N, and P

In the four water fractions, we measured the total C, total N, and total P concentrations. The total and filtered (dissolved) fractions of the organic carbon (TOC and DOC) and nitrogen (TN and DN) were measured in a Total Organic Carbon Analyzer (TOC-V CPN Shimadzu). The different fractions of particulate organic carbon in the filters were measured using the solid sample module (SSM-5000 A) of the TOC analyzer. Particulate nitrogen and phosphorus on the filters and total phosphorus (TP) in the liquid samples were measured after potassium persulfate digestion (Carmouze, 1994). After digestion, the TN was measured in the TOC-V CPN (Shimadzu), whereas the TP was measured spectrophotometrically (SP2000UV) by the ascorbic acid method (Mackereth et al., 1978). All filters were combusted (550°C for 4 h) prior to filtration and after the filtration and before the analysis were oven dried (60°C for >24 h) and weighed.

All bulk seston, bacteria and dissolved C:N, C:P, and N:P ratios were calculated on a molar basis (mol: mol).

### Bacterial Density and Metabolism

The BD was estimated in the bulk, <1.6 and <0.7 μm fractions via flow cytometry, which allowed the determination of the number of cells in each fraction and the number of cells retained on the filters. The samples were fixed with buffered formaldehyde (4% final concentration) and kept at -80°C until analysis in a BD FACSCalibur flow cytometer (within 5–8 months). The cells were stained with Syto 13 and the cytograms analyzed in the FlowJo X 10.0.7r2 software (Gasol and Del Giorgio, 2000; Sarmento et al., 2008).

Bacterial production (BP) was measured via the [^3^H]-leucine incorporation and microcentrifugation method (Smith and Azam, 1992), incubating bulk water samples for 2.5 h. We assumed the molar percentage of leucine in the protein pool was equal to 0.073, the intracellular isotopic dilution was equal to 2 and a carbon:protein ratio of 0.86.

Bacterial respiration rates (BR) were measured in the <1.6 μm filtered water by following the oxygen consumption in 6 mL exetainer vials without headspace in the dark over variable periods of time (incubations were terminated when O_2_ concentrations were ≤8% of initial O_2_ concentrations; from 12 to 72 h of incubation). The oxygen concentrations were measured with a gold tip microprobe connected to a OXY-Meter (UNISENSE) and were converted to carbon using a respiratory quotient = 1.0 (Briand et al., 2004).

Specific bacterial production and respiration (BP and BR cell^-1^) were calculated by dividing the respiration rates from each lake by the number of cells in the bulk and <1.6 μm fractions, respectively. The bacterial carbon demand was calculated as the sum of BP and BR (Alonso-Sáez et al., 2007).

### Statistical Analysis

The log_10_ of the absolute concentrations of C, N, and P (μM) were linearly regressed against each other and compared to the expected Redfield ratios (106:16:1) by ANCOVA. Significant interactions indicated differences in slopes and significant intercepts indicated differences in the magnitude of the ratios.

The log_10_ of the C:N:P ratios of seston, bacteria and the dissolved fractions and of BP, BP cell^-1^, BR, BR cell^-1^, BGE (BP/[BP + BR]) and BCD (BP + BR) were linearly regressed against conductivity in order to assess the effect of residence time on the variation of these ratios, assuming conductivity as a proxy of water residence time (Rennella and Quirós, 2006; Anderson and Stedmon, 2007).

The log_10_ of the BD, BP, BR, BGE, and BCD were also linearly regressed against log_10_ of the bacterial C:N, C:P, and N:P ratios in order to determine whether the bacterial internal stoichiometry was coupled to bacterial metabolism. Conversely, the bacterial C:N, C:P, and N:P ratios were linearly regressed against potential substrates (seston and dissolved) C:N, C:P, and N:P ratios, respectively, in order to determine whether the substrate stoichiometry of these pools were correlated.

All statistics were carried out in R 3.2.2. (R Core Team, 2016) and all regressions assumptions were tested with the *gvlma* package (Pena and Slate, 2014).

## Results

The C:N:P ratios were variable for all fractions with means across all lakes being higher than expected by the classical Redfield ratio (mean seston C:N:P = 771:59:1; mean bacterial C:N:P = 320:34:1; mean dissolved C:N:P = 1225:173:1). Considering the extreme values, the lowest ratios were found in the bacterial fraction (90:19:1) and the highest in the dissolved fraction (1225:173:1; **Table 2**).

**Table 2:** Molar ratios of carbon (C), nitrogen (N) and phosphorus (P) for bacteria and the three substrates fractions (bulk, seston and dissolved) for the 15 lakes along a trophic gradient.

Lakes	Bacterial per cell			Bulk (Seston + <1.6 μm)			Seston (>1.6 μm)			Dissolved (<0.7 μm)		
	(femtomol:femtomol)			(μmol: μmol)			(μmol: μmol)			(μmol: μmol)		
	C:N	C:P	N:P	C:N	C:P	N:P	C:N	C:P	N:P	C:N	C:P	N:P
Bonfim	20.4	1675.3	82.1	11.5	156.3	13.6	8.1	431.7	53.1	14.6	104.0	7.1
EAJ	5.0	286.2	57.0	14.9	766.5	51.4	13.0	1502.7	115.2	17.1	1938.3	113.5
Extremoz	3.2	89.9	28.0	10.3	811.6	78.8	9.7	653.2	67.3	11.7	382.7	32.8
Arituba	12.3	289.1	23.5	9.6	430.0	44.8	16.2	915.3	56.6	7.3	311.1	42.7
Carcará	10.2	342.0	33.5	11.6	554.0	47.7	14.8	779.4	52.6	11.0	490.8	44.7
Jiqui	13.9	262.5	18.9	1.1	247.3	228.4	19.9	683.4	34.4	0.6	121.8	197.7
Lagoa Azul	8.1	395.6	48.9	1.5	902.8	584.4	13.5	2134.4	157.6	0.5	389.1	805.6
Jambeiro	2.8	108.7	39.1	13.1	1113.4	85.2	14.7	651.6	44.4	12.8	811.7	63.6
Ilhota	11.8	447.6	37.8	13.9	502.2	36.1	22.6	1350.8	59.9	10.2	332.3	32.7
Cruzeta	8.0	212.9	26.6	10.9	350.5	32.0	10.1	233.7	23.2	11.8	444.1	37.7
“Caliman”	7.4	148.7	20.1	15.2	563.6	37.1	19.2	681.8	35.6	12.9	801.1	61.9
Boqueirão	5.2	163.7	31.3	10.5	1632.5	156.1	7.8	614.7	79.2	11.1	6259.8	565.5
Gargalheiras	5.7	116.1	20.4	10.2	654.0	64.4	7.5	226.0	30.2	11.9	1886.7	158.7
Traíras	6.3	164.0	26.3	9.3	1057.2	114.2	8.0	456.2	57.0	10.0	2925.4	293.6
Dourado	9.3	220.1	23.6	12.0	565.1	47.1	11.3	244.2	21.5	8.6	1175.3	136.1
Mean	8.5	319.9	34.5	10.4	687.1	108.1	13.1	770.6	59.2	10.1	1224.9	172.9
Min.	2.8	89.9	18.9	1.1	156.3	13.6	7.5	226	21.5	0.5	104	7.1
Max.	20.4	1675.3	82.1	15.2	1632.5	584.4	22.6	2134.4	157.6	17.1	6259.8	805.6

**FIGURE 2:**
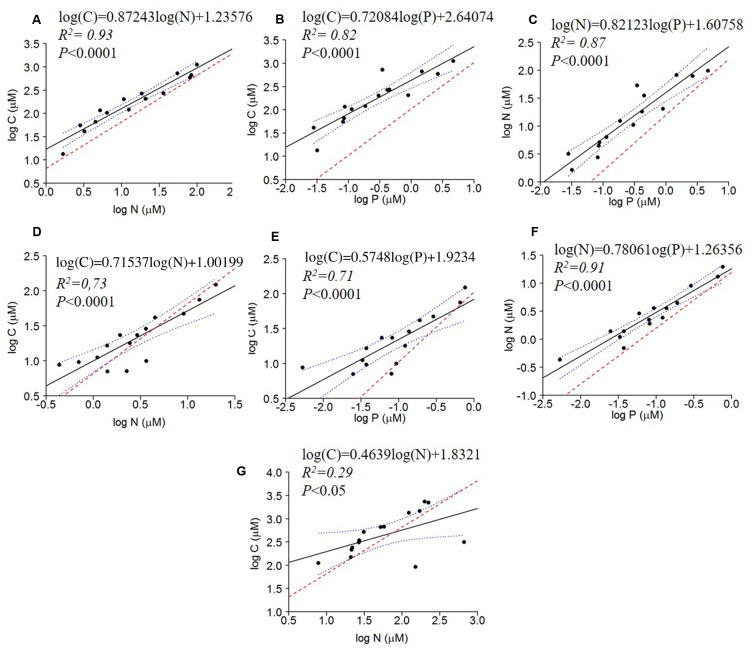
Regressions of log of the absolute concentrations of C × N, C × P and N × P of seston **(A–C)**, bacterial **(D–F)** and dissolved fractions **(G)** compared to the Redfield ratio (dashed red line). The regression fit is indicated by black solid lines and 95% confidence intervals by blue dotted lines.

All bivariate regressions between the absolute concentrations of C, N, and P of all fractions were positive and significant (C to N; C to P; N to P), except for C × P and N × P of the dissolved fraction (*P* > 0.05) (**Table 3**). The comparison via ANCOVA of these relationships with those expected by the Redfield ratio revealed differences for all fractions with the exception of the dissolved C × P and N × P. All seston measured slopes and the bacterial C × P and N × P slopes were higher than Redfield ratios, whereas the bacterial C × N and the dissolved C × N slopes were lower than Redfield. The intercepts of these regressions were also significantly higher than zero, except for seston and bacterial C × N, the bacterial N × P and the dissolved C × P and N × P intercepts (**Table 3** and **Figure 2**).

**Table 3:** Comparison of slopes and intercepts between measured and expected Redfield relationships of pairs of nutrients (carbon, C, nitrogen, N and phosphorus, P) for seston, bacterial and dissolved fractions for the 15 lakes along the trophic gradient.

	C × N		C × P		N × P	
	Measured	*P*-value	Measured	*P*-value	Measured	*P*-value
**Seston**						
*slope*	**9.22**	**<0.01**	**218.11**	**<0.01**	**22.80**	**<0.05**
*intercept*	37.19	>0.05	**136.24**	**<0.05**	**11.39**	**<0.05**
**Bacteria**						
*slope*	**5.74**	**<0.05**	**135.31**	**<0.05**	**23.39**	**<0.0001**
*intercept*	4.04	>0.05	**6.82**	**<0.05**	0.51	>0.05
**Dissolved**						
*slope*	**1.06**	**<0.001**	554.4	>0.05	68.96	>0.05
*intercept*	**611.99**	**<0.05**	308.2	>0.05	65.69	>0.05

*The expected Redfield slopes are 106 for C × P, 6.625 for C × N and 16 for N × P whereas the expected Redfield intercepts are equal to 0. Significantly different from Redfield slopes and intercepts are highlighted in bold*.

**FIGURE 3:**
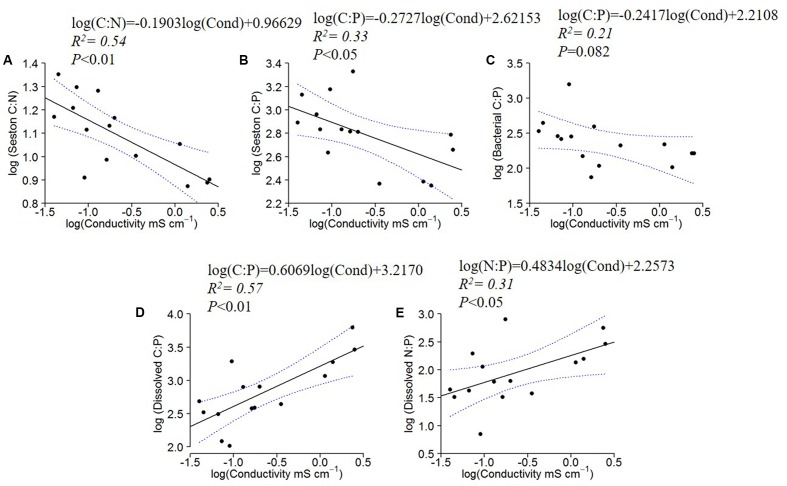
Log_10_ linear regressions of seston C:N and C:P against specific conductivity **(A,B)**, bacterial C:P against specific conductivity **(C)** and dissolved C:P **(D)** and N:P ratios **(E)** against specific conductivity (residence time). The regression fit is indicated by black solid lines and 95% confidence intervals by blue dotted lines.

The seston C:N and C:P ratios decreased significantly with water conductivity, and the bacterial C:P followed the same pattern, although with a marginally insignificant relationship (*P* = 0.082) (**Figures 3A–C**). The dissolved organic C:P and N:P, however, increased with increasing conductivity (**Figures 3D,E**). All other regressions of C:N:P of the fractions (Seston N:P, Bacterial C:N and N:P, and dissolved organic C:N) with conductivity were not significant (not shown).

**FIGURE 4:**
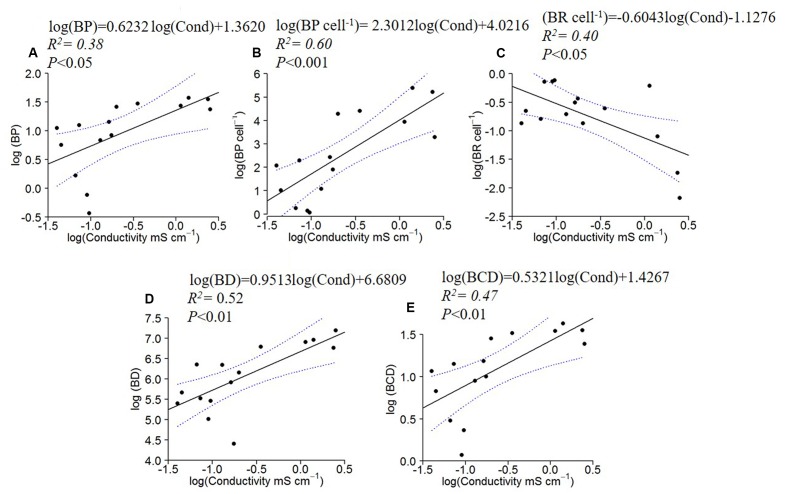
Log_10_ linear regressions of bacterial production (BP; **A**), specific bacterial production **(B)** and specific bacterial respiration **(C)**, bacterial density (BD; **D**) and carbon demand (BCD; **E**) against specific conductivity. The regression fit is indicated by black solid lines and 95% confidence intervals by blue dotted lines.

BP, BP cell^-1^, BD and BCD increased with specific conductivity (**Figures 4A,B,D,E**), while BR cell^-1^ displayed the opposite pattern, decreasing with conductivity (**Figures 4C**).

**FIGURE 5:**
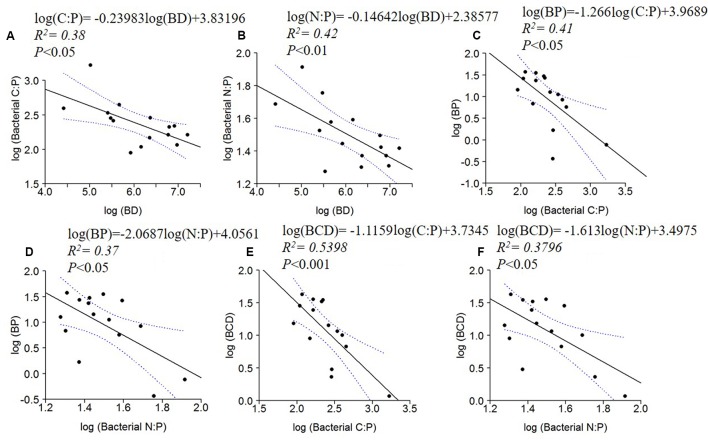
Log_10_ linear regressions of ratios of bacterial C:P **(A)** and N:P **(B)** against bacterial density (BD), BP against bacterial C:P **(C)** and N:P **(D)** and BCD against bacterial C:P **(E)** and bacterial N:P **(F)**. The regression fit is indicated by black solid lines and 95% confidence intervals by blue dotted lines.

BD, BP and BCD were negatively correlated to bacterial C: P and N: P ratios (**Figure 5**). No significant relationships were found between BR or BR cell^-1^ and bacterial C:N:P ratios.

**FIGURE 6:**
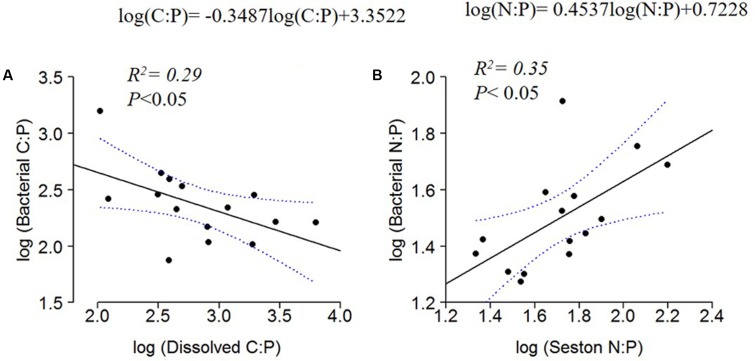
Log_10_ linear regressions of bacterial C:P against dissolved C:P **(A)** and bacterial N:P against seston N:P ratio **(B)**. The regression fit is indicated by black solid lines and 95% confidence intervals by blue dotted lines.

The C:N:P ratios in the dissolved organic pool and the seston pool had contrasting relationships when regressed against bacterial ratios. Bacterial C:P decreased with dissolved C:P (**Figure 6A**), whereas bacterial N:P increased with seston N:P (**Figure 6B**).

## Discussion

As expected for inland waters, the C:N:P ratios in the low-latitude lakes surveyed in the current study were mostly higher than the Redfield ratios. Nonetheless, seston ratios became more similar to Redfield ratios with increasing conductivity, with decreased C:N and C:P ratios. We argue that specific conductivity is a useful proxy for WRT in these systems and therefore, longer WRT may provide microbiota greater opportunity to extract nutrients from dissolved organic materials. Consistent with this argument, we observed positive correlations between C:P and N:P in the dissolved fraction (i.e., DOC:TDP and TDN:TDP) with increasing conductivity (**Figure 3**). We also observed faster specific growth rates in systems with higher conductivity (**Figure 4**), suggesting that part of the explanation for higher P content in systems with increased conductivity may have been the GRH (Sterner and Elser, 2002; Makino et al., 2003). Thus, we confirmed our hypothesis that the increasing WRT corresponded with decreased C:N and C:P in seston and bacterial biomass.

Our observations for freshwater seston stoichiometry in the tropics were similar to the mean seston estimates from a survey of 226 lakes from primarily temperate North America, and including some lakes from Europe, Africa, and Asia (Elser et al., 2000a). The mean seston C:N:P in this synthesis was 307:30:1, which is similar to the ratio that we observed in the bacterial size fraction (mean C:N:P 320:35:1) but lower than the mean values we observed in seston (C:N:P 607:108:1; **Table 2**). The highest seston C:N and C:P values were found at the lowest specific conductivities, perhaps indicating a strong terrigenous influence on the seston in the lower conductivity systems (from watershed input by rainfall).

Because our results were based on field data, numerous factors could have been affecting the stoichiometric behavior in these different lakes. Certainly, temperature is one factor that can affect seston and biomass stoichiometry (Cotner et al., 2006; Martiny et al., 2013; Phillips et al., under review) but we observed no significant differences in temperatures along the conductivity gradient (**Table 1**). However, there were differences in rainfall across the conductivity gradient that we observed, with greater rainfall occurring in the eastern, more ocean-influenced region and less in the semi-arid, inland region (as description in the study area section). Our observations were made when this region was being influenced by El Nino which was one of the factors that contributed to the semi-arid region lakes experiencing an unusual prolonged drought and drastically reducing their volumes (Medeiros et al., 2015; Costa et al., 2016; Mendonça et al., in press).

Therefore, it seems more likely that the patterns we observed between dissolved and particulate stoichiometry and specific conductivity may have been coupled to changes in residence times among the different systems. Few studies have examined relationships between water residence times and seston stoichiometry in freshwaters. One that did, however, in the Experimental Lakes of Canada (ELA), found that longer residence times correlated positively, not negatively as we found, with C:N:P (Hecky et al., 1993). Differences may have arisen from the fact that in Hecky et al.’s (1993) study a small number of lakes (<10) were included and only lakes with very short residence times (<3 months) were compared to longer residence time systems (>6 months). Also, the lakes were not influenced by a precipitation gradient and even the long WRT lakes were oligotrophic. On the other hand, the semi-arid area lakes in our study were all human-made reservoirs built in a way that the water outflow is only possible when large inputs of water enables dam overflow, and with WRT usually greater than 24 months.

The overall pattern is a bit more complicated in freshwaters due to differences in source material that are often coupled to differences in stoichiometry. Terrestrial stoichiometric signatures tend to be higher than microbial biomass signatures due to increased concentrations of structural carbohydrates and lignin (Elser et al., 2000a; Schlesinger and Bernhardt, 2013). In lakes with short residence times, the input of terrestrial material is high and tends to dominate the organic matter composition (Cole et al., 2002; Pace et al., 2004; Wilkinson et al., 2013). However, at longer residence times, there is less input of these components and increased influence from aquatic microbes and aquatic plants. Due to more rapid growth of aquatic plants and microbes in aquatic systems and lower amounts of structural material, their stoichiometry tends to be richer in N and P (lower C:N and C:P) than terrestrial plants and they also degrade at faster rates (Enriquez et al., 1993), which means that long residence time systems should have nutrient signatures that reflect this. That seems to be the case of the lakes in the semiarid region.

Perhaps relatedly, Kellerman et al. (2014) recently demonstrated that the composition of DOM was strongly correlated with water residence times in boreal lakes. Short residence time systems had DOM composition that reflected the surrounding watershed but as residence times increased, the terrestrial signature was increasingly lost. In these boreal systems, organic matter in the longer residence time systems showed increased N content. The N-containing DOM compounds were either tightly recycled or resistant to decomposition processes (Kellerman et al., 2015). Our observation of increased TDN:TDP (i.e., N:P in the dissolved fraction) ratios with specific conductivity may reflect this N enrichment (**Figure 3E**), but it is also likely that there was tight recycling of both N and P due to the limiting nature of these elements in lakes as DOC:TDP (i.e., C:P in the dissolved fraction) also increased with specific conductivity (**Figure 3D**).

The decreasing seston C:P, C:N and bacterial C:P with increasing conductivity indicated P enrichment of the organisms with increasing WRT (**Figure 3**). This enrichment was concomitant with increasing dissolved C:P and N:P ratios, which suggests that the plankton may have been ‘mining’ P from this dissolved pool. Although this enrichment of the particulate pool via the dissolved pool was not sufficient to bring the seston ratios to values similar to Redfield ratios, it does illustrate the central mechanism envisioned by Redfield through which planktonic organisms can modify their environment given that they are provided with sufficient residence time to process that material (Redfield, 1934; 1958). Nutrients that are not incorporated into biomass are ‘discarded’ to the sediments or lost to the atmosphere in the oceans. Similar dynamics are likely occurring in all lakes, but the effects are more obvious at longer residence times.

The fact that bacterial N:P ratios were positively correlated with seston N:P ratios was consistent with other studies showing a tendency for bacterial biomass to reflect some of the same stoichiometric tendencies of the larger plankton pool. In a survey of lakes in the Midwest of the United States, Cotner et al. (2010) demonstrated that the bacterial pool had stoichiometry similar to the Redfield ratio with the total seston pool showing a similar pattern, but with slightly higher C:N, C:P, and N:P ratios. Higher N and P content in the microbial pool is often attributed to higher growth rates in these organisms (Bratbak and Dundas, 1984; Makino et al., 2003; Godwin et al., 2016), but somewhat surprisingly, the stoichiometry of the bacterial and seston pools do not often differ a great deal, perhaps due to the fact that they are drawing from the same dissolved and particulate nutrient pools and/or that large portions of the microbial community are dormant (Lennon and Jones, 2011).

Alternatively, the bacteria could be responding positively to the seston pool due to nutrients that are being provided directly from the phytoplankton. The seston in many of these lakes is dominated by cyanobacteria and zooplankton (rotifers and copepods, Eskinazi-Sant’Anna et al., 2007). Among the cyanobacteria, several N-fixing species like *Cylindrospermopsis raciborskii*, *Anabaena circinalis*, *Aphanizomenon gracile* (Fonseca et al., 2015; Medeiros et al., 2015), *Microcystis aeruginosa* and *Oscillatoria* sp. (Chellappa and Costa, 2003) have been reported to occur and dominate the phytoplankton community of these eutrophic lakes, i.e., the high WRT. The appearance/dominance of cyanobacteria in the phytoplankton community can have a strong impact in seston stoichiometry, with a tendency of lowering C:N ratios. Also, the release of dissolved organic N-based compounds could be an important source of both organic matter and N for bacteria in these lakes, especially semi-arid ones during El Niño events when there are very limited external organic matter inputs (Glibert and Bronk, 1994).

Another factor contributing to lower stoichiometric ratios in the longer residence time systems could be changes in the bacterial community composition. Although we did not explicitly address this issue, BD and bacterial production both increased along the conductivity gradient, perhaps reflecting an increased influence of relatively rapidly growing organisms (**Figure 4**). Nonetheless, there was no significant relationship between the seston-POC: bacterial-POC ratio and conductivity, suggesting that similar changes in the larger seston community may have also been occurring along the gradient. The GRH hypothesizes that rapidly growing organisms should increase their RNA and ribosome content (Elser et al., 2000b; Makino et al., 2003). Because RNA is P-rich relative to the mean composition of organisms, rapidly growing organisms should be more P-rich, with decreased C:P and N:P ratios. Recently, Godwin et al. (2017) demonstrated that the relative growth rate also plays an important role in this dynamic. Although it is difficult to assess the relative growth rate of organisms in natural environments, the increase in bacterial production and cell specific production that we observed (**Figure 4**) is consistent with the idea that growth rates and relative growth rates increased with increasing conductivity.

Relatedly, Goldman et al. (1979) argued that phytoplankton in the open ocean are growing at or near maximal rates because the C:P and C:N ratios are near Redfield ratios. They argued that the long residence time open ocean is much like a chemostat in that the biomass does not change much over time, yet there can be variation in nutrient supply that is drawn down by the biomass that is present with the main loss being grazing. Despite fundamental differences between open ocean and lakes, semi-arid lakes become more similar to open ocean during extended drought periods when there is negligible input of allochthonous organic matter and the WRT increases. These lakes will likely have more variable biomass concentrations over time (still to be tested) due to greater variation in the supply rates due to episodic events such as changes in mixing depths, pulses due to storms, etc. Nevertheless, our results suggest that the mechanism envisioned by Redfield may be universal for all aquatic ecosystems under appropriate conditions.

Even though we observed a strong effect of WRT on bacterial and seston stoichiometry in these lakes, other factors not addressed here and that may co-vary with changes in environmental conditions may also be important. Grazing pressure on bacteria by protists is stronger on larger, fast dividing cells (Sherr et al., 1992), thus potentially favoring smaller, relatively more carbon rich cells (Norland, 1993). Also, weaker top–down effects of zooplankton have also been found to favor low-quality phytoplankton, with important potential repercussions for food webs (Hessen et al., 2005).

## Conclusion

Our observations indicated WRT can be an important factor affecting the stoichiometry of plankton in freshwater ecosystems. As WRT increased, the biomass stoichiometry of seston and the bacterial size-fraction became more enriched with N and P. The dissolved organic nutrient pool stoichiometry demonstrated a negative correlation with residence time, suggesting that organic N and P pools may have been important sources of nutrients in long residence time systems. Water residence time is a parameter that has been little explored in freshwaters with respect to stoichiometry and needs to be considered in the future studies.

## Author Contributions

NT Performed field and lab work and writing. AA planned, performed field and lab work and contributed to the writing. JC planned, performed field and lab work and contributed to the writing.

## Conflict of Interest Statement

The authors declare that the research was conducted in the absence of any commercial or financial relationships that could be construed as a potential conflict of interest.

## Footnotes

1 http://www.bom.gov.au/climate/enso/\#tabs=SOI
