# Supplementary material for: Redfield Ratios in Inland Waters: Higher Biological Control of C:N:P Ratios in Tropical Semi-arid High Water Residence Time Lakes
Source: Front Microbiol. 2017 Aug 8;8:1505. doi: 10.3389/fmicb.2017.01505 (PMC5551281; doi:10.3389/fmicb.2017.01505)
Supplement: Supplementary file 1 [file Table_1.DOCX]

Supplementary Material

Redfield ratios in inland waters: higher biological control of C:N:P ratios in tropical semi-arid high water residence time lakes

*** Correspondence:** Corresponding Author: amado@ufrnet.br

Supplementary Table 1. Heterotrophic and autotrophic bacterial abundance and heterotrophic/autotrophic ratio in all size fractions.

| **Bacterial density (cells mL^-1^)** | | | | | | | | |
| --- | --- | --- | --- | --- | --- | --- | --- | --- |
| **Heterotrophic (H)** | | | **Autotrophic (A)** | | | **H/A ratio** | | |
| Bulk | <1.6 | <0.7 | Bulk | <1.6 | <0.7 | Bulk | <1.6 | <0.7 |
| 5.61E+06 | 1.97E+06 | 8.09E+05 | 2.84E+05 | 9.59E+04 | 2.67E+03 | 20 | 21 | 303 |
| 1.52E+07 | 1.10E+07 | 3.22E+06 | 7.48E+05 | 1.53E+05 | 3.23E+03 | 20 | 72 | 998 |
| 8.04E+06 | 1.04E+06 | 1.04E+06 | 9.08E+04 | 8.91E+02 | 5.94E+02 | 88 | 1170 | 1754 |
| 7.85E+06 | 5.00E+06 | 1.17E+06 | 1.40E+06 | 4.96E+05 | 8.70E+03 | 6 | 10 | 134 |
| 7.49E+04 | 3.60E+04 | 3.68E+04 | 2.92E+04 | 1.00E+04 | 1.14E+03 | 3 | 4 | 32 |
| 1.70E+05 | 1.85E+05 | 8.89E+04 | 8.43E+04 | 7.41E+04 | 3.24E+03 | 2 | 2 | 27 |
| 2.23E+06 | 8.92E+05 | 4.07E+05 | 1.25E+04 | 1.26E+03 | 7.39E+04 | 178 | 706 | 6 |
| 2.30E+06 | 6.42E+05 | 5.99E+05 | 2.05E+03 | 4.50E+04 | 3.83E+04 | 1123 | 14 | 16 |
| 4.01E+05 | 3.40E+05 | 7.97E+04 | 6.41E+04 | 3.01E+04 | 2.76E+03 | 6 | 11 | 29 |
| 7.08E+05 | 2.59E+05 | 2.14E+04 | 1.27E+05 | 2.97E+03 | 1.43E+03 | 6 | 87 | 15 |
| 2.53E+04 | 3.33E+05 | 1.39E+05 | 5.29E+02 | 4.51E+04 | 1.16E+04 | 48 | 7 | 12 |
| 5.45E+06 | 1.01E+06 | 9.38E+05 | 7.70E+05 | 3.59E+04 | 2.61E+04 | 7 | 28 | 36 |
| 3.05E+05 | 1.75E+05 | 5.20E+04 | 3.50E+04 | 3.42E+03 | 9.20E+02 | 9 | 51 | 57 |
| 2.55E+05 | 1.82E+05 | 1.06E+05 | 3.99E+04 | 2.99E+04 | 8.16E+03 | 6 | 6 | 13 |
| 1.28E+06 | 1.22E+06 | 3.55E+05 | 1.63E+05 | 6.21E+04 | 4.41E+02 | 8 | 20 | 806 |

**
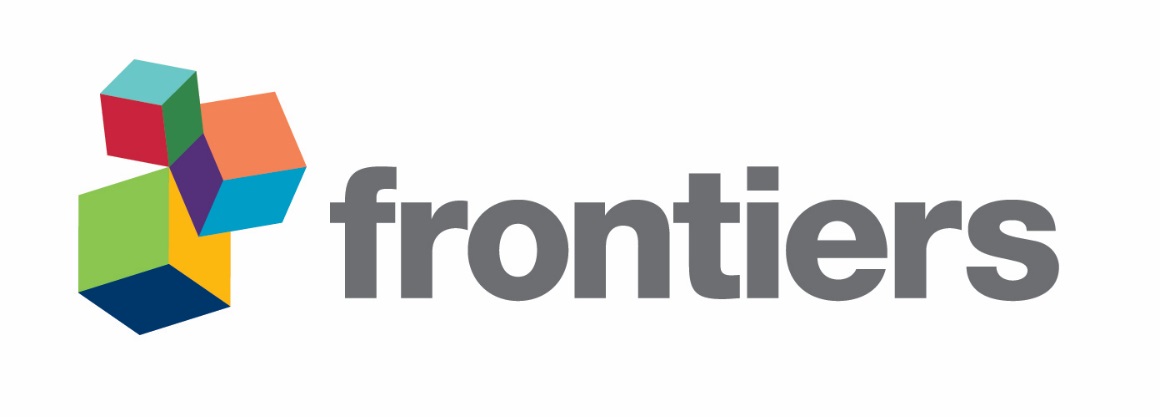
**
